# Microalbuminuria and Glomerular Filtration Rate in Paediatric Visceral Leishmaniasis

**DOI:** 10.1155/2013/498918

**Published:** 2013-06-23

**Authors:** Neena Verma, Chandra Shekhar Lal, Vidyanand Rabidas, Krishna Pandey, Dharmendra Singh, Sanjay Kumar, Rakesh Bihari Verma, Pradeep Das

**Affiliations:** ^1^Rajendra Memorial Research Institute of Medical Sciences (Indian Council of Medical Research), Agam Kuan, Patna 800 007, India; ^2^Laboratory of Clinical Biochemistry, Rajendra Memorial Research Institute of Medical Sciences (Indian Council of Medical Research), Agam Kuan, Patna 800 007, India

## Abstract

Visceral leishmaniasis, caused by *Leishmania donovani*, is a serious form of leishmaniasis and fatal if untreated. Nearly half of the VL cases are children. There are very few studies of renal function in pediatric visceral leishmaniasis. The aim of this study was to evaluate renal dysfunction by studying glomerular filtration rate (GFR), microalbuminuria, and microscopic examination of urine. Laboratory analysis was performed on blood and urine samples of 40 parasitologically confirmed pediatric VL cases. Laboratory data of urine examination showed albuminuria in 10% (4/40), white blood cells in 20% (8/40), hematuria in 10% (4/40), microalbuminuria in 37.5% (15/40), and decreased GFR in 27.5% (11/40). Renal involvement was manifested in most of the pediatric VL cases. These findings may help clinicians in decision making for safe and suitable antileishmanial treatment particularly in childhood VL.

## 1. Introduction

The leishmaniases, an infectious disease endemic in tropical, Asian, and southern European countries, is caused by obligate intramacrophage protozoa and is transmitted through the bite of infected female sandflies. The disease phenotypes include visceral leishmaniasis (VL), post-kala-azar dermal leishmaniasis (PKDL), cutaneous leishmaniasis (CL), and mucosal leishmaniasis (ML). Visceral leishmaniasis (VL), caused by *Leishmania donovani*, is the most serious, mainly affects children, and is fatal if untreated. Nearly half of the VL cases occur in children (childhood or paediatric VL) [1]. The disease is prevalent over large areas of Bihar, India. VL is a febrile illness that is characterized by weight loss, pancytopenia, hepatosplenomegaly, and lymphadenopathy and can be complicated by acute renal damage [2–4]. Even though kala-azar nephropathy is still poorly understood, kidney dysfunction in this disease has been reported in several studies. Immune complex deposition, T cells, and adhesion molecules activation have been shown to be important mechanisms of injury in glomerulonephritis occurring in visceral leishmaniasis [5–9]. 

One of the first human studies about renal involvement in kala-azar was a cohort study of 50 patients with visceral leishmaniasis [10]. Proteinuria and/or microscopic haematuria or pyuria was observed in 51% of patients. Urinary protein excretion was elevated in 57% of patients, and in all of them, this was <1 g/24 h. Renal function was evaluated in 11 patients with visceral leishmaniasis, and results stated that 5 patients presented macroscopic haematuria and 1 developed acute nephritic syndrome [11], while urine examination showed proteinuria in 10 patients and haematuria and leukocyturia in 7 and 6 cases, respectively. In a cross-sectional study of 50 patients with chronic visceral leishmaniasis, a decreased glomerular filtration rate (GFR) in 28% of the cases is reported [12]. More recently, the presence of increased albumin excretion in 44% of patients [13] was observed. The glomerular damage may manifest as mesangioproliferative or membranoproliferative glomerulonephritis due to depositions of immune complexes in the glomeruli. Moreover, tubulointerstitial nephritis may occur and consists of tubular degeneration and inflammatory infiltration with renal impairment [2, 5, 14–16]. Acute kidney injury had been observed in children with visceral leishmaniasis [17]. Paediatric VL patients are found more in numbers in Bihar, India, and studies examining nephrotoxicity in children are scarce.

 Conventional amphotericin B (AmB) is the treatment of choice for antileishmanial treatment in Bihar, India. This drug possesses high antileishmanial efficacy, but it is associated with a high risk of renal toxicity in addition to other side effects (rigor, fever, malaise, anorexia, thrombophlebitis, and bone marrow suppression) [18]. In addition to the poor selectivity of amphotericin B between human cholesterol and fungal ergosterol, other mechanisms seem to be involved in the pathogenesis of renal toxicity of the drug [19]. In India, liposomal amphotericin B showed lower rates of toxicity than conventional amphotericin B or amphotericin B lipid complex [20–23]. Hence, keeping in mind the selectivity of amphotericin B with human cholesterol and hypocholesterolemia in paediatric visceral leishmaniasis [24], we examined pediatric VL patients for glomerular involvement before start of AmB treatment. This will be the first ever information about renal injury in pediatric VL patients from this part of India.

Kidney disease is mostly silent, common and harmful but treatable. The only reason for requesting creatinine is to assess kidney function. Routine reporting of estimated glomerular filtration rate (GFR) alongside creatinine has proved to be a useful tool for clinicians in the detection and management of kidney injury. On the other hand, microalbuminuria (MA) is considered as an early marker of glomerulonephritis and is defined as a persistent elevation of albumin in urine of 30–300 mg/day. 

 The aim of our study is to determine the prevalence of renal injury in paediatric VL patients of Bihar, India, who are mostly being treated in the endemic areas by AmB and evaluate it according to GFR and microalbuminuria for the purpose of early detection with regards to renal injury.

## 2. Materials and Methods

### 2.1. Patients and Samples

 The observational study included pediatric VL patients aged between 2 and 14 yrs that admitted to the indoor ward of Rajendra Memorial Research Institute of Medical Sciences (Indian Council of Medical Research) Patna, India, for antileishmanial treatment by Amphotericin B deoxycholate. Altogether, 40 subjects were selected for this study after obtaining informed consent from each patient's next of kin. The serum, fresh urine and 24-hour urine, samples were collected from 40 confirmed paediatric VL patients microscopically positive for *Leishmania donovani *parasite in splenic/bone marrow aspirate before onset of antileishmanial treatment. Urine analysis, serum creatinine, and urea were measured in all patients. The samples were kept in refrigerator (2–8°C) until assayed.

### 2.2. Urine Analysis

Urine analysis was done on a fresh morning sample of urine within one hour of collection in a sterile dry container. Physical examination for colour, appearance, and any sediment formation was done. Urine was mixed properly and tested by URiSCAN urine strips (YD Diagnostics, Republic of Korea) for chemical examination, that is, bilirubin, urobilinogen, ketone, protein (albumin), nitrite, glucose, pH, specific gravity, leukocytes, and blood as per the procedure mentioned in the leaflet insert.

### 2.3. Microscopic Examination of Urine Sample

About 5 mL of properly mixed urine sample was centrifuged in a centrifuge tube for 5 min at 2,500 rpm. Supernatant was poured off, and one drop of shaked deposit was placed on a glass slide for microscopic examination under low and high power objectives (10–15 fields) for the presence of white blood cells (WBCs), erythrocytes (RBCs), casts (granular, hyaline, cellular, etc.), epithelial cells, crystals, and so forth per high power field.

### 2.4. Estimated Glomerular Filtration Rate (eGFR) Measurement

eGFR was measured in children using Schwartz formula. This employs the serum creatinine (mg/dL), the child's height (cm), and a constant to estimate the glomerular filtration rate:

(1) $$\begin{array}{c} \text{eGFR}=\frac{k\times \text{Height}}{\text{Serum}\;\text{Creatinine}}, \end{array}$$

where *k* is a constant that depends on muscle mass. For infants and children of age 1 to 12 years, *k* = 0.55.

### 2.5. Microalbuminuria Measurement

A 24-hour urine sample was collected from the study subjects in clean plastic containers with boric acid as preservative. The urine samples were kept in the refrigerator till analysis. A commercial turbidometric kit (Tulip Diagnostic, Goa, India) was used for the measurement of MA. The company's instructions were strictly adhered to. One mL of reagent buffer (polyethylene glycol, Tris/HCL buffer, and sodium chloride) was pipette into seven different test tubes (6 tubes for standard, 1 test tube for sample), and 0.1 mL of standards (human serum albumin) with different concentrations (5, 10, 20, 50, 100, and 200 *μ*g) and 0.1 mL of urine samples were added to the test tubes. The tubes were thoroughly mixed, and the initial absorbance was read spectrophotometrically against 340 nm. Antihuman albumin antibody (0.1 mL) was added; the tubes were thoroughly mixed and incubated for 30 minutes. The final absorbance of each solution was read spectrophotometrically at 340 nm, and the concentrations were calculated from the constructed standard curve.

### 2.6. Serum Creatinine and Urea Measurement

Serum creatinine and urea were assayed according to Jaffe Kinetic and Urease-GLDH Kinetic methods following protocol of commercial kit (LABKIT, Spain), respectively.

Ethics approval was obtained from ethics committee of the institute and was in accordance with the 1975 Helsinki Declaration on Human Rights, as revised in Edinburgh 2000.

## 3. Results

**Figure 1:**
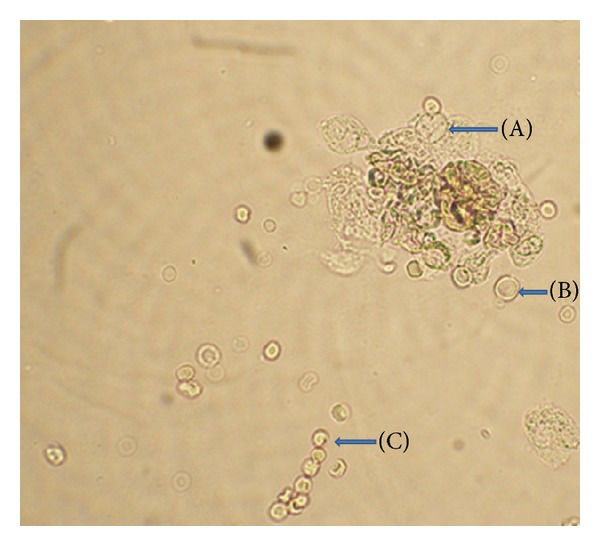
Microscopy of urinary sediments showing (A) epithelial cells, (B) RBCs, and (C) WBCs under high power objective (×40x).

All of the study patients revealed within normal range serum urea and creatinine levels at the time of admission. The routine urinalysis showed albuminuria in 10% (4/40) and increased pus cells (WBCs) in 20% (8/40) of VL cases before treatment with AmB (Figure 1). Hematuria was observed in 10% (4/40) patients. However, 15/40 (37.5%) of the study pediatric VL patients had detectable MA before treatment. The estimated GFR (eGFR) ranged between 60 and 89 mL/min/1.73 m^2^ in 27.5% (11/40) pediatric VL patients before treatment. The normal reference range of eGFR in paediatric group ranged between 92.3 and 122.5 mL/min/1.73 m^2^.

## 4. Discussion

Amphotericin B nephrotoxicity in children is a known complication. We investigated renal impairment in children suffering from VL before onset of Amphotericin B. In Sudanese VL patients, the renal injuries are mostly of glomerular in nature. The presence of this renal abnormality could be attributed to infiltration by infected macrophages and/or deposition of immune complexes in the glomeruli. The present study showed that urinary albumin excretion could be predictive for the development of *de novo* renal function impairment in pediatric VL cases.

 The presence of significant microalbuminuria in 37.5% of VL patients with normal serum urea and creatinine was indicative of renal glomerular damage. However, in our present study, normal serum urea and creatinine levels probably point to the crudeness of these tests as indicators of renal damage. Another cross-sectional study of Lima Verde et al. on 50 VL patients has reported decreased GFR in 28% of the cases, and our study reported decreased GFR in 27.5% of pediatric VL patients which indicates evidence of renal damage [12]. These abnormalities in glomerular filtration, urinary concentration, and acidification might be consistently associated with the chronic form of VL. Further, decreased GFR could be related to fluid loss, hypotension, and immunological glomerular damage. Since in this part of India the treatment is mostly being done by AmB in disease-endemic districts and drug combination therapy for VL is the trend worldwide, more sensitive investigations like eGFR, MA, and thorough microscopic examinations are needed to differentiate from early renal damage before start of chemotherapy.

In conclusion, glomerular involvement is the main renal injury in pediatric VL patients. The eGFR, MA, and microscopic examination maybe helpful in prediction of early renal damage. These parameters can provide an understanding of kidney injury and help treating doctors in decision making for safe and suitable antileishmanial treatment in pediatric group of patients.
